# Severe dysphagia after a posterior cervical spine fusion at the C1–C3 level and its improvement after correction surgery for malalignment: a case report

**DOI:** 10.1186/s13256-022-03505-5

**Published:** 2022-07-18

**Authors:** Siravich Suvithayasiri, Payothorn Decharin

**Affiliations:** 1 Department of Orthopedics, Chulabhorn Hospital–Chulabhorn Royal Academy, Bangkok, Thailand; 2 Department of Neurological Surgery, Chulabhorn Hospital–Chulabhorn Royal Academy, Bangkok, Thailand

**Keywords:** Dysphagia, Trismus, Posterior cervical instrumentation, Upper cervical spine, Case report

## Abstract

**Background:**

The development of dysphagia and trismus following posterior C1–C3 fusion is rare compared to occipitocervical fusion, and there are very few reports in the literature.

**Case presentation:**

A 75-year-old Thai man who had an extradural tumor at the C1/C2 level developed severe dysphagia and trismus immediately after tumor resection and C1–C3 fusion. During the surgery for malalignment correction, the C1–C2 screws were re-aligned into an increased lordotic position. The symptoms resolved immediately post-surgery.

**Conclusions:**

Short upper cervical fusion can produce trismus and dysphagia in a relatively flexed position. Preoperative planning should be individualized based on the patient’s resting neutral alignment to prevent complications.

## Background

Dysphagia and trismus have been reported on a number of occasions as severe complications after posterior occipitocervical fusion [1–3]. The atlantooccipital joint contributes to most of the flex and extended movement of the cervical spine. Surgical fixation and malalignment are believed to be the leading causes of physical obstruction of pharyngeal space and therefore impairment of the swallowing process [4, 5]. Reports on dysphagia and trismus after surgery are scarce in the clinical setting of short cervical fusion that does not involve the occipital bone (for example, C1–C2 fusion). We report the case of a 75-year-old man who developed severe dysphagia and trismus immediately after C1–C3 fusion surgery, which improved dramatically following surgery for malalignment correction.

The Institutional Review Board (IRB) of the Chulabhorn Royal Academy approved this study. The patient provided informed consent. All processes were performed following standard regulations, and the CARE guidelines were followed [6].

## Case presentation

**Fig. 1:**
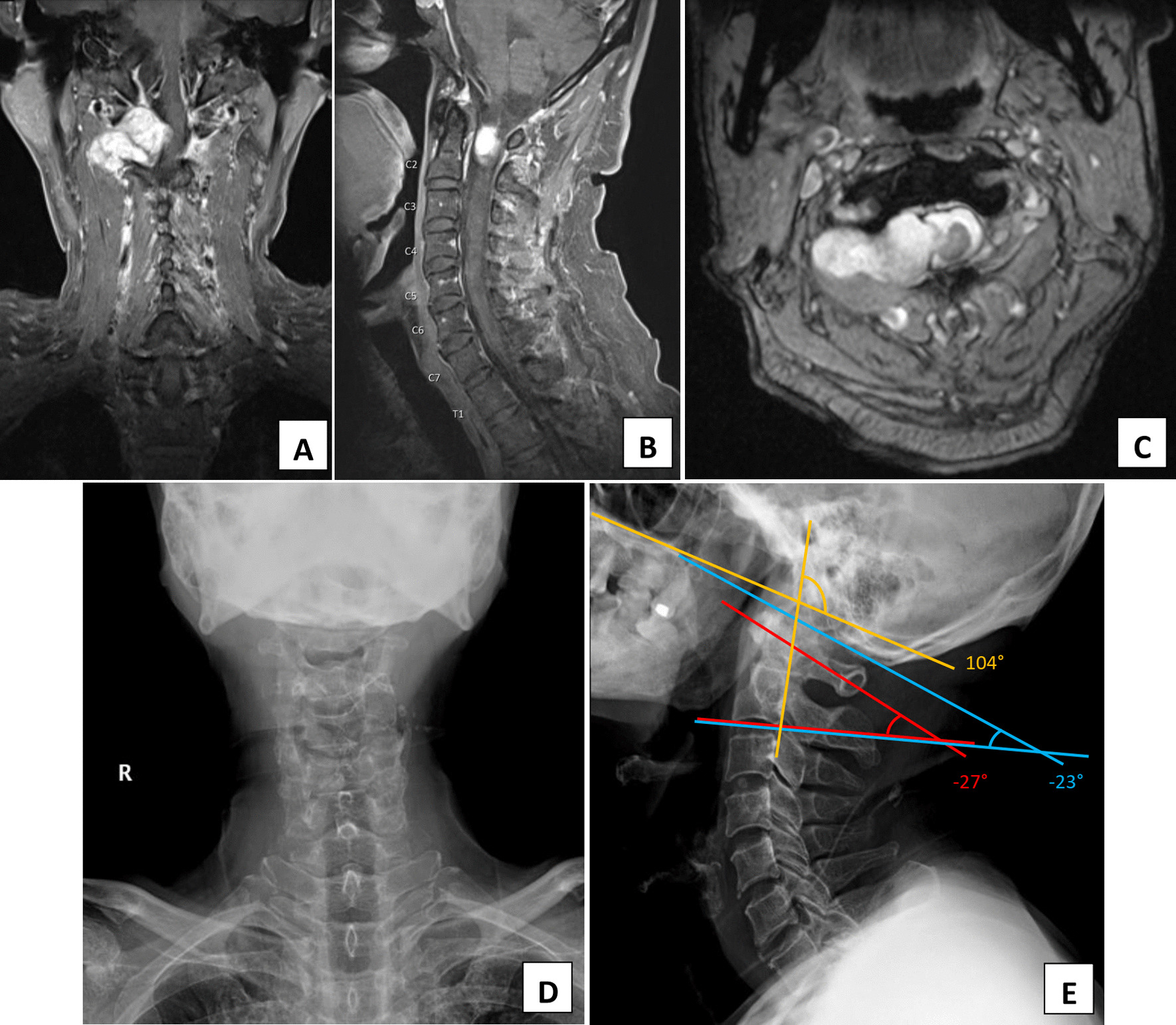
T1-weighted, post-contrast injection magnetic resonance imaging images show an enhancing lesion at an extradural location with an intradural extension at the right side of the C1/C2 vertebrae in the coronal (**a**), sagittal (**b**), and axial (**c**) plane. The mass effect caused corresponding spinal cord compression with left posterolateral displacement. No obvious cord signal abnormality was seen. Plain film readiographs of the preoperative cervical spine in the anteroposterior (**d**) and lateral view (**e**) also show the measured angle of occiput-C2 (blue), C1–C2 (red), and Takami’s angle (yellow) of − 23°, − 27°, and 104°, respectively

A 75-year-old Thai man with a recent history of tuberculosis infection presented with an enlarged tonsil. Following an initial physical examination, the otolaryngologistsn sent the patient for computed tomography (CT) scan of his neck, which revealed that his tonsil was normal but that there was a mass located incidentally at his cervical spine at the level of C1/C2. There was no history of neck pain, gait abnormalities, or neurological problems. Physical examination revealed only increased tendon reflex of his upper and lower extremities. His neck range of motion was full, and his bowel bladder function was normal. Neither paralysis nor paresthesia was observed. The magnetic resonance imaging (MRI) revealed an extradural enhancing mass at the right C1/C2 level compressing the spinal cord (Fig. 1). The provisional diagnosis was a benign tumor with suspicion of schwannoma. Despite the subtle clinical neurological deficit, the MRI finding of severe spinal cord compression was concerning, and treatment options were discussed. Due to the high risk of creating instability following resection of the tumor, the treatment chosen was that of additional fusion of the upper cervical spine. Preoperative plain radiographs of the cervical spine and the upper cervical sagittal alignment are shown in Fig. 1.

A standard posterior midline cervical spine approach was carried out. Since preoperative imaging showed that both C2 and C3 pedicles were feasible enough to insert the pedicular screws, we decided to use the technique to achieve maximal strength and stability of the construct system assisted by the CT navigation system. The pedicle screw insertion at the C2 level was uneventful. However, lateral breeching was encountered during the insertion on the right side at the C3 level. Concerned for the risk of bilateral vertebral artery injuries, we switched to the lateral mass screw technique on the other side of C3. Intraoperative neuromonitoring using motor-evoked potentials) and somatosensory-evoked potentials were used and remained unchanged from baseline throughout the surgery.

After surgery, and while still in the recovery room, the patient complained of discomfort in his throat, accompanied by difficulty swallowing and opening his mouth. Bedside swallowing evaluation revealed some weakness of the neck muscles and desaturation following swallowing of a small amount of water (3 ml). He could not voluntarily cough sharply. The gag reflex was positive on both sides but delayed. Therefore, the rehabilitation physicians suggested keeping him with a nasogastric tube until further evaluation.

**Fig. 2:**
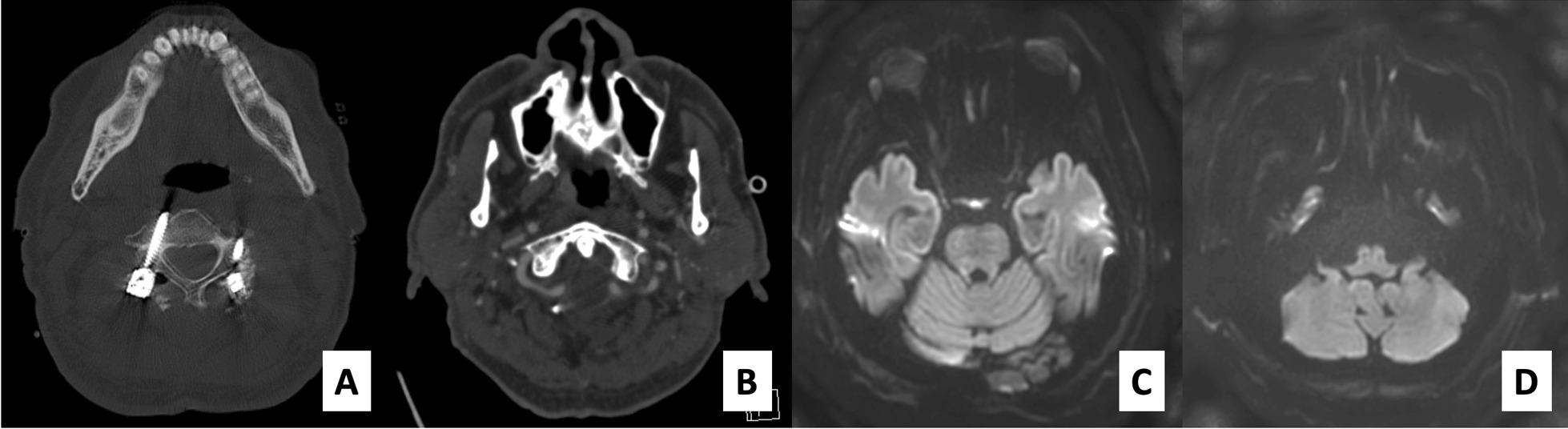
Computer tomography reveals the right C3 pedicular screw trajectory, which involves the medial border of the right foramen transversarium and left C3 lateral mass screw (**a**). Postoperative computed tomography angiography demonstrated a patent of right vertebral artery and good distal flow (**b**). Postoperative brain magnetic resonance imaging with diffusion-weighted imaging technique did not show any diffusion restriction (**c**, **d**)

**Fig. 3:**
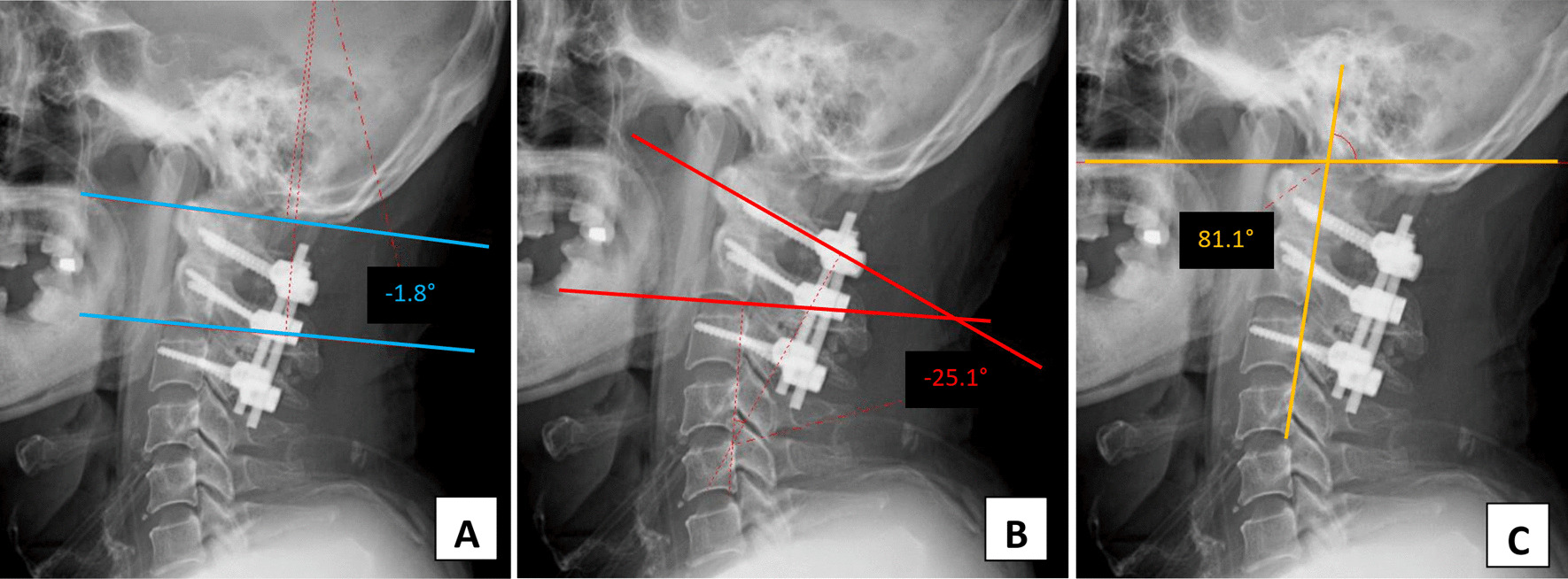
Plain radiographs after the first operation reveal the neutral alignment of the upper cervical spine region. The occiput-C2 angle is − 1.8° (**a**), the C1–C2 angle is − 25.1° (**b**) and the craniovertebral junction angle proposed by Takami is 81.1° (**c**)

Postoperative radiographic examination illustrated that the screw-rod system was intact and that the atlantoaxial alignment was neutral. CT angiography showed no VA injury with good distal flow. MRI of the brain did not demonstrate any evidence of stroke or suspected cause of dysphagia (Fig. 2). Using the McGregor line as a reference of the occipital bone, the occiput-C2 and C1–C2 angles were − 1.8° and − 25.1°, respectively (the minus value indicates that the angle is in lordosis). The craniovertebral angle proposed by Takami *et al.* [5] to evaluate the craniovertebral fixation angle, namely, the angle between the line parallel to the bony palate and the posterior longitudinal line of vertebrae of C2, was 81.1°. The space between the posterior border of the mandible and the anterior of the cervical spine was narrowed, as was the distance between the screw-rod fixation system and the occiput (Fig. 3). Taken together, these findings led us to believe that the cause of the trismus and dysphagia was the position of the screws. Therefore, an operation to adjust the position of the C1 and C2 screws to be relatively more extended position was performed on the eighth day after the first surgery.

**Fig. 4:**
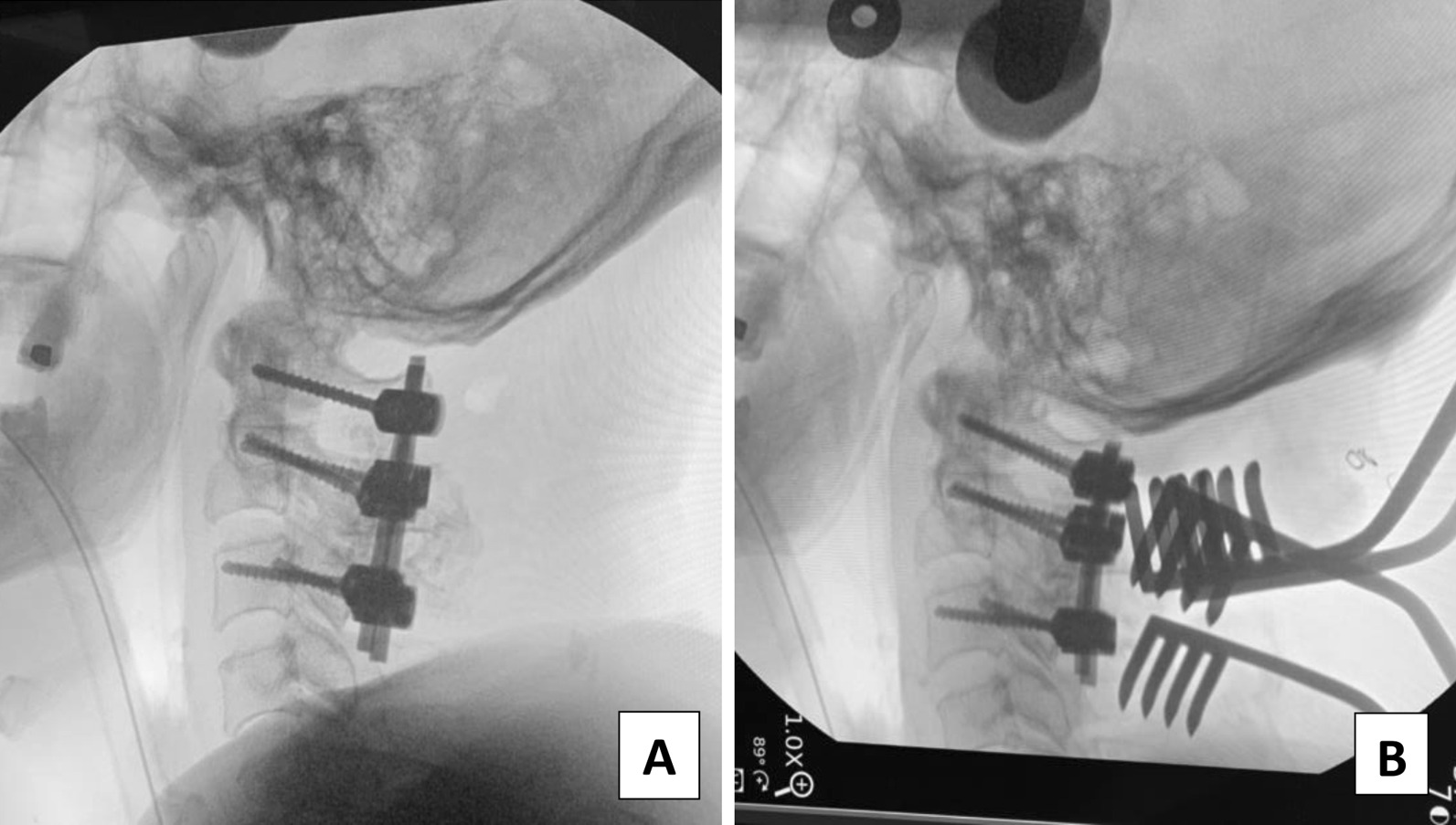
Intraoperative fluoroscopic images showing pre-adjustment (**a**) and post-adjustment (**b**) status of the screw-rod fixation system. Post-adjustment, the distance between both sides of the C1 and C2 screw head was shortened (as well as the length of both rods), thus creating the C1/C2 alignment in a relatively more lordotic position.

**Fig. 5:**
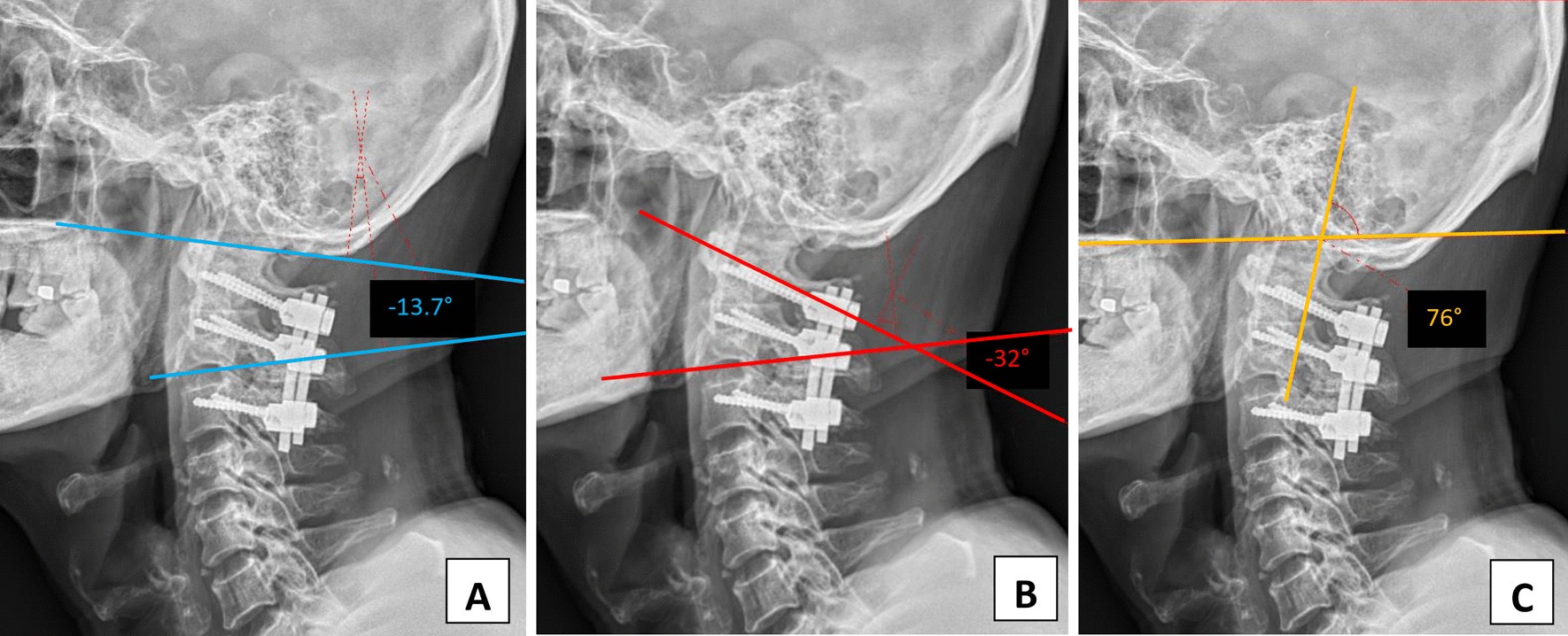
Plain radiographs after the second operation show an extended alignment of the upper cervical spine region. The post-correction occiput-C2 angle was − 13.7° (**a**), the C1–C2 angle was − 32° (**b**) and the craniovertebral junction angle proposed by Takami was decreased to 76° (**c**)

Comparison of intraoperative fluoroscopic images before and after adjustment of the implant positions revealed that following the adjustment, the alignment of the C1–C2 level was more extended (Fig. 4). The space of interest behind the mandible associated with the cervical spine and just beneath the occipital bone associated with the screw-rod system was widened. Immediately after the revision surgery, the patient’s trismus and dysphagia symptoms improved drastically. He could swallow 3 and 10 ml of water in a test without coughing or havein g a wet voice. He passed the cup drink test of 30 ml water by straw without choking. As the segment was more lordosis, the occiput-C2 and C1–C2 angles were changed to − 13.7° and − 32°, respectively. At the same time, the craniovertebral angle of Takami was decreased to 76° (Fig. 5).

## Discussion

Dysphagia after the posterior instrumented fusion of the upper cervical spine is believed to be caused by limited of range of motion of the upper vertebrae and obstruction of the pharyngeal space from exceedingly flexed posture [4, 5]. According to Panjabi *et al.*, the range of motion of the C1–C2 level is only 10.9° compared to 21° of the occiput-C1 level [7]. Therefore, the fixation of C1–C2 or C3 level (sparing the occiput) should be less likely to result in postoperative dysphagia or trismus.

**Fig. 6:**
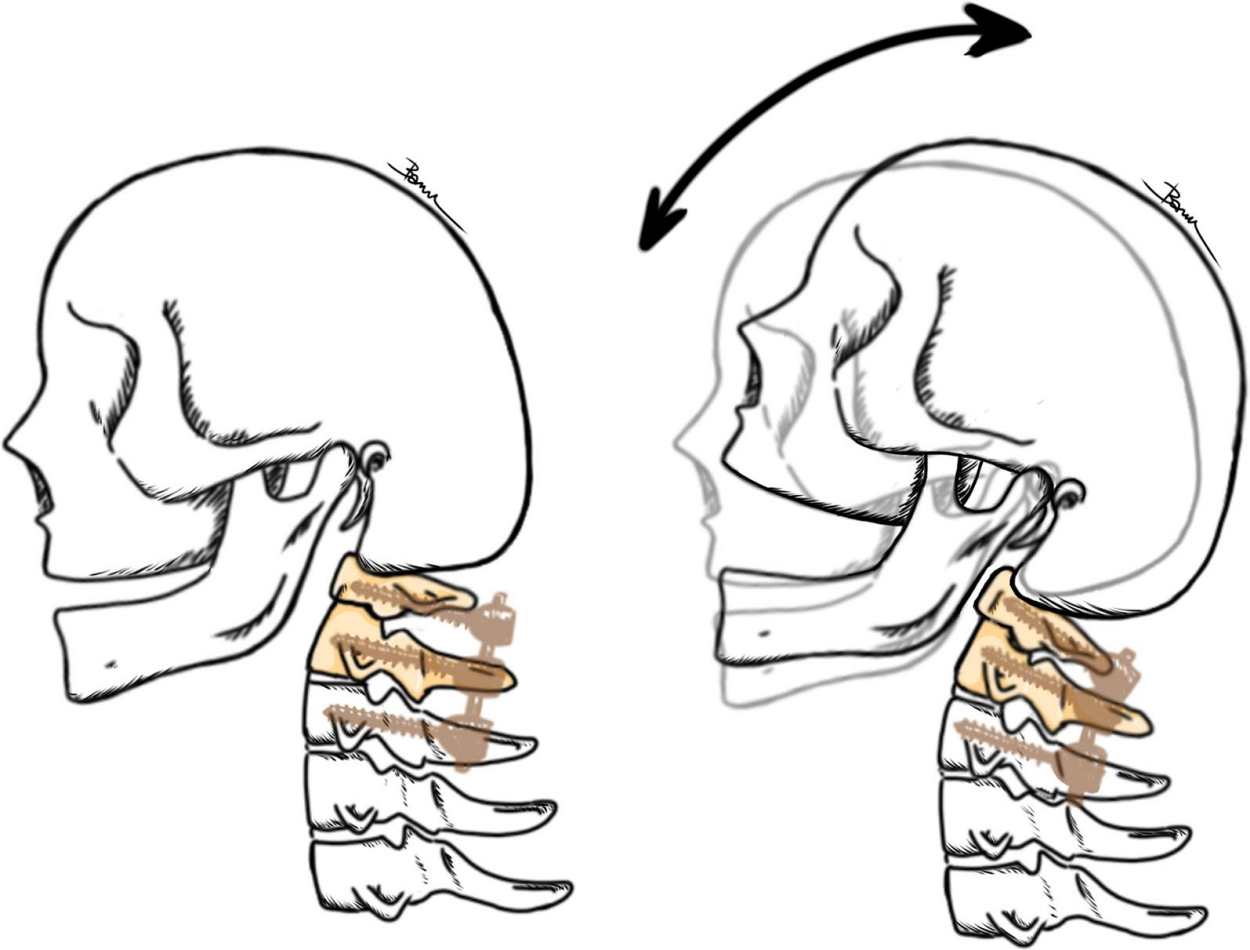
An illustration shows the possible mechanism of dysphagia after upper cervical spine fixation without involvement of the occipital bone. A larger soft tissue space between the C1 posterior arch and the occiput is achieved as the C1–C2 angle is more natural, or lordosis. This could improve trismus and dysphagia by giving the skull, the mandible and the pharynx more space to be move

However, our case is a rare complication of dysphagia and trismus following instrumentation of the C1–C3 level, which resolved after revision surgery with increased lordosis angle of the C1–C2 level. The result strongly supports the notion that even if the occiput segment was spared, this sagittal mismatch could still be the etiology of dysphagia. The mechanism could be the increased kyphosis angle of the C1–C2 levels and the decrease in soft tissue space between the C1 posterior arch and the occiput, which resulted in a more flexed position and further limited the range of motion of the skull. Moreover, there was limited space for the mandible and pharynx to move. As a result, trismus and dysphagia developed (Fig. 6). This case could raise surgeons’ concerns about the importance of sagittal alignment.

To the best of our knowledge, only two studies have reported similar findings. Bekelis *et al.* reported that a 61-year-old man encountered severe dysphagia after a C1–C3 posterior cervical fusion surgery [8]. The patient’s upper cervical spine alignment was not discussed in this report and revision surgery was not carried out. The authors concluded that the this case was complicated by neurogenic dysphagia associated with vagal nerve dysfunction [8]. Misawa *et al.* reported the case of a 69-year-old man who developed dysphagia and trismus immediately after C1–C2 fixation surgery [9]. The C1–C2 alignment was revised into a more lordotic position 12 days after surgery, and his symptoms disappeared immediately following revision surgery. These authors also suggested that these complications may occur even after short upper cervical spine fixation without the occiput. Furthermore, the hyperflexion position of the neck could cause the screw heads to contact the occipital bone and consequently interrupt the extended motion, causing dysphagia [9].

To prevent this occurrence, the alignment goal should be planned before surgery. Takami *et al.* proposed a simple measurement method of the craniovertebral junction preoperatively and used it as a postoperative goal. The mean angle in regular healthy volunteers was 99.9° (range: 85°–118°) [5]. However, as in our case, this angle varied at each time point (104° before surgery, 81.1° after the first surgery and 76° after correction). Therefore, the inconsistency of this angle makes it questionable.

On the other hand, the classic occiput-C2 angle was more reliable. In our case, the occiput-C2 angle was − 23° before surgery and decreased to − 1.8° after the first operation, when the patient developed dysphagia. After surgical revision, a more lordosis angle was achieved at − 13.7°, closer to the preoperative value. The symptoms disappeared promptly, suggesting that the preoperative occiput-C2 angle could be used as an alignment goal to prevent complications. However, the cutting point is yet to be determined. This means that even if the occiput is not fused, manipulating a short segment of the C1–2 angle can still cause dysphagia and trismus.

Numerous studies in asymptomatic volunteers have also reported a range in the C1–C2 angle from − 20.8° to − 54° [10–16]. Although we attempted to keep the C1–C2 angle in the normal range in the first operation (25.1°), the patient still developed complications. This result suggests that the goal of the C1–C2 angle should not be based on ranges from the literature but should be tailor-made and based on each patient’s anatomical neutral C1–C2 angle. However, this would not be easy in some situations as reduction or overcorrection is sometimes needed [17–19]. Future study is necessary to identify individualized occiput-C2 and the C1–C2 allowance angles, based on their neutral position, and determine just how much deviation of this angle can be tolerated before dysphagia or trismus results.

## Conclusion

We report here rare complications of severe dysphagia and trismus secondary to C1–C3 posterior instrumented fusion that occurred even when fusion of occipital bone was spared. The malalignment of C1–C2 fixation in the kyphosis position could result in the flexed position, resulting in the patient developing dysphagia. Surgical correction increasing the lordotic position of C1–C2 could solve the problem. Prevention strategy relies on preoperative planning and should be based on the neutral position of each patient’s sagittal profile of the upper cervical spine.

## Data Availability

The datasets used and/or analyzed during the current study are available from the corresponding author on reasonable request.

## Abbreviation

C: Cervical spine level
